# Cell Entry and Trafficking of Human Adenovirus Bound to Blood Factor X Is Determined by the Fiber Serotype and Not Hexon:Heparan Sulfate Interaction

**DOI:** 10.1371/journal.pone.0018205

**Published:** 2011-05-26

**Authors:** Stéphanie Corjon, Gaëlle Gonzalez, Petra Henning, Alexei Grichine, Leif Lindholm, Pierre Boulanger, Pascal Fender, Saw-See Hong

**Affiliations:** 1 University Lyon 1, INRA UMR 754, Retrovirus and Comparative Pathology, Lyon, France; 2 Department of Microbiology and Immunology, University of Göteborg, Institute for Biomedicine, Göteborg, Sweden; 3 Institut Albert Bonniot, CRI INSERM-UJF U-823, La Tronche, France; 4 Got-a-Gene AB, Kullavik, Sweden; 5 Unit for Virus-Host Interaction, UMI-3265, CNRS-EMBL-UJF, Grenoble, France; University of Minnesota, United States of America

## Abstract

Human adenovirus serotype 5 (HAdV5)-based vectors administered intravenously accumulate in the liver as the result of their direct binding to blood coagulation factor X (FX) and subsequent interaction of the FX-HAdV5 complex with heparan sulfate proteoglycan (HSPG) at the surface of liver cells. Intriguingly, the serotype 35 fiber-pseudotyped vector HAdV5F35 has liver transduction efficiencies 4-logs lower than HAdV5, even though both vectors carry the same hexon capsomeres. In order to reconcile this apparent paradox, we investigated the possible role of other viral capsid proteins on the FX/HSPG-mediated cellular uptake of HAdV5-based vectors. Using CAR- and CD46-negative CHO cells varying in HSPG expression, we confirmed that FX bound to serotype 5 hexon protein and to HAdV5 and HAdV5F35 virions via its Gla-domain, and enhanced the binding of both vectors to surface-immobilized hypersulfated heparin and cellular HSPG. Using penton mutants, we found that the positive effect of FX on HAdV5 binding to HSPG and cell transduction did not depend on the penton base RGD and fiber shaft KKTK motifs. However, we found that FX had no enhancing effect on the HAdV5F35-mediated cell transduction, but a negative effect which did not involve the cell attachment or endocytic step, but the intracellular trafficking and nuclear import of the FX-HAdV5F35 complex. By cellular imaging, HAdV5F35 particles were observed to accumulate in the late endosomal compartment, and were released in significant amounts into the extracellular medium via exocytosis. We showed that the stability of serotype 5 hexon∶FX interaction was higher at low pH compared to neutral pH, which could account for the retention of FX-HAdV5F35 complexes in the late endosomes. Our results suggested that, despite the high affinity interaction of hexon capsomeres to FX and cell surface HSPG, the adenoviral fiber acted as the dominant determinant of the internalization and trafficking pathway of HAdV5-based vectors.

## Introduction

The human adenovirus (HAdV) capsid is composed of eleven well identified structural proteins, of which the hexon is the major component with 240 copies forming the 20 facets and 30 edges of the icosahedral capsid. The penton is the second most represented capsid protein, with 12 copies of penton located at each apex. Each penton capsomere is made up of a fiber, a triple beta-stranded fibrous protein [1], anchored to a pentameric protein, the penton base, closing up the vertices of the icosahedron (reviewed in [2], [3]). HAdVs are divided into subgroups or species A to F, covering 51 different serotypes. The members of species C (HAdV2, HAdV5) and species B (HAdV3, HAdV35) are the most studied and characterized in terms of capsid structure, cell entry mechanisms, cellular response and gene transfer (reviewed in [2], [3]). The classical cell entry and trafficking pathway of HAdV5, as demonstrated by epithelial cell models of adenoviral infection *in vitro*, consists of (i) the fiber binding to CAR, the Coxsackie B and Adenovirus Receptor [4]–[11], followed by (ii) the interaction of the penton base RGD motifs with the cellular integrins alpha_V_beta3 and alpha_V_beta5, [12]–[15], which promotes virus endocytosis into clathrin-coated vesicles [16], [17]. In step (iii), partially uncoated HAdV5 particles are released from the early endosomal compartment into the cytosol, a process involving the capsid protein VI [18], [19]. (iv) Dynein and microtubules mediate the cytoplasmic transit of the residual HAdV5 capsid, which docks at the nuclear pore complex [20], [21], before (v) the nuclear import of the viral nucleoprotein core [22], [23].

Contrasting with the vast scientific information available on HAdV5 and its multiple interactions with host cell components, the clinical application of HAdV5, the most widely utilised serotype as gene transfer vector, has suffered from several drawbacks. The prevalence of anti-HAdV antibodies in the human population results in the rapid neutralisation of HAdV5 vector after *in vivo* administration. Secondly, intravenous delivery of HAdV5 vector results in the liver uptake of the vast majority of the virus particles, and therefore do not reach their target cells or tissues. Numerous strategies have been employed to overcome these hurdles, notably by engineering mutant or chimeric vectors to evade the neutralising antibodies and ablate the hepatotropism of the vector, but the results have been somewhat disappointing (reviewed in [24]).

Recent breakthrough in the HAdV-host interactions showed that the vector particle accumulation in the liver is the result of HAdV5 binding to human blood coagulation factor X (FX) via the hexon capsomeres, followed by the interaction of the HAdV5-FX complexes to heparan sulfate proteoglycan (HSPG) molecules which are present in high concentration at the surface of Kupffer cells [25]–[34]. Further dissection of the molecular mechanism of the HPSG-mediated cellular uptake of HAdV5-FX complex revealed the importance of *O*- and *N*-sulfation of HPSG in this high affinity pathway, and the requirement of alpha_V_ integrins as secondary receptors for an efficient internalization step [35]. In contrast to HAdV5, it has been observed *in vivo* that HAdV35 vectors have liver transduction efficiencies which are of four orders of magnitude lower than that of HAdV5 vectors [36]. Likewise, fiber-pseudotyped or chimeric fiber-carrying HAdV5 vectors showed less hepatotropism, compared to HAdV5. This was the case for HAdV5F35, which carried serotype 35 fibers [37], HAdV5/35 chimeric vector, which carried serotype 35 fiber knob domains [38], and HAdV5F2/BAdV4, carrying chimeric human-bovine fibers [39]. Intriguingly however, HAdV5, HAdV5F35 and HAdV5F2/BAdV4 vectors were all composed of the serotype 5 hexon capsomere, thus suggesting the contribution of factors other than hexon, FX and HSPG to the mechanism of liver uptake of FX-HAdV5 complex *in vivo*. Both HAdV35 and the HAdV5F35 chimera use CD46, one of the cell attachment receptors recognized by subspecies B HAdVs besides desmoglein-2 [40], and to a lesser degree HSPG molecules, to infect epithelial cells [41]–[44].

In the present study, we sought to determine the influence of capsid proteins other than the hexon, viz. penton base and/or fiber, on the interaction of FX with HAdV5-based vectors, and their FX- and HSPG-mediated cell entry pathway and gene transduction. To this aim, we analyzed the binding of wild type HAdV5 (HAdV5wt), penton base or fiber mutants of HAdV5, and fiber 35-pseudotyped HAdV5 vector (HAdV5F35) to heparan sulfate in the presence or absence of FX by surface plasmon resonance *in vitro*. We analyzed the effect of FX on the HAdV5wt- and HAdV5F35-mediated transduction of CHO cells expressing HSPG (CHO-K1), the alternative receptors for HAdV5 [45]–[47] and HAdV35 viruses [41]–[44], and HSPG-negative CHO cells (CHO-2241). Both cell lines lack the CD46 and CAR receptors for HAdV5F35 and HAdV5, respectively.

We found that FX bound to HAdV5 hexon protein *via* its Gla-domain, and enhanced the binding of HAdV5wt and HAdV5F35 vector particles to surface-immobilized hypersulfated heparin (HS) *in vitro*, and to cellular HSPG *in vivo*. We also found that FX augmented the efficiency of cellular transduction by HAdV5, but had a negative effect on the transduction by HAdV5F35. Our experimental data showed that this negative effect did not involve the cell attachment or the endocytic step of the FX-HAdV5F35 complex, but the intracellular trafficking. In the presence of FX, the HAdV5F35 particles accumulated in the late endosomal compartment, resulting in a delay in their vesicular release and nuclear import. Furthermore, HAdV5F35 were released in significant amounts in the extracellular medium *via* exocytosis, resulting in lower numbers of particles reaching the nucleus. Our results suggested that the serotype 35 fiber determined the cell internalization and trafficking pathway of the HAdV5F35 vector, despite the absence of known fiber receptor at the plasma membrane, and acted dominantly despite the interaction between hexon and cell surface HSPG mediated by FX. This observation has significant implications for the future design of target tissue-redirected adenoviral vectors.

## Results

### Gla domain-dependence of FX-mediated binding of serotype 5 hexon protein and adenovirions to heparan sulfate in vitro

**Figure 1:**
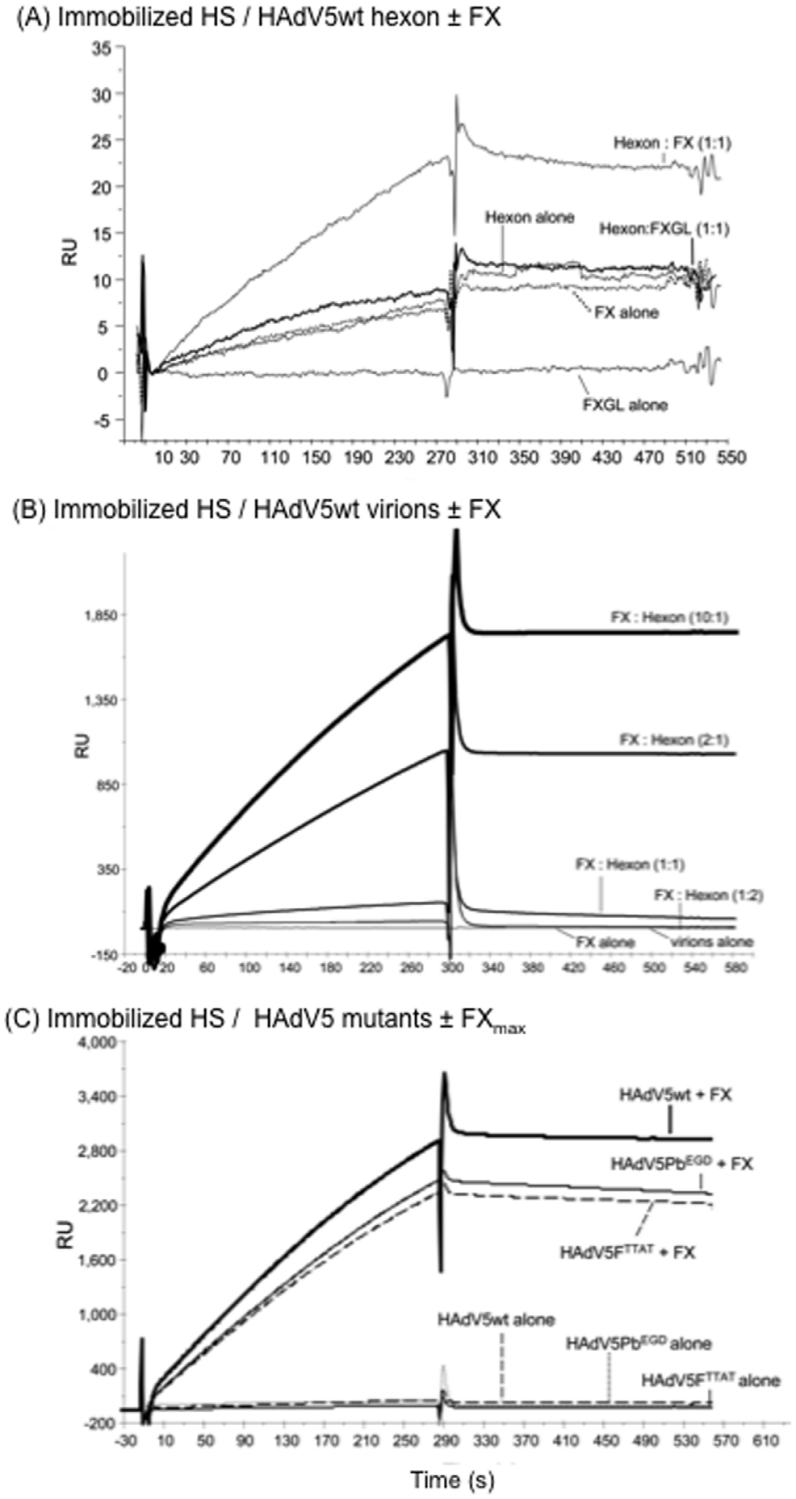
SPR analysis of the *in vitro* binding of HAdV5 hexon capsomeres and HAdV5-based vectors to immobilized HS with or without factor X (FX) bridging. Representative sensorgrams for (**A**) HAdV5 hexon capsomeres alone, or with FX or Gla domainless FXGL, (**B**) HAdV5wt virions alone or with FX, and (**C**) HAdV5 virion mutants HAdV5F^TTAT^ and HAdV5Pb^EGD^ alone or with FX. In (A) and (B), the molecular ratio of FX to hexon protein (isolated capsomeres as in (A), or virion-encapsidated hexons, as in (B)) is indicated in parenthesis. The control sensorgrams with FX and FXGL alone were obtained at FX and FXGL concentrations of 8 µg/ml, corresponding to the concentration in human adult serum (FX_max_). In (C), FX was also used at 8 mg/ml. Hexon capsomeres, HAdV5wt virions and HAdV5F^TTAT^ and HAdV5Pb^EGD^ mutants bound to immobilized HS only in the presence of FX. RU, response units.

The interaction between soluble HAdV5wt hexon protein and heparan sulfate *in vitro*, directly or indirectly *via* FX, was investigated using surface plasmon resonance (SPR) analysis and a hypersulfated form of heparin (HS), recognized as the best structural model to mimic the heparan sulfate chains contained in HSPG [48], [49]. HS was covalently immobilized onto the biosensor chip, and the binding of hexon to HS was assessed using FX in a stoichiometric ratio of 1∶1 with hexon protein. A truncated version of FX devoid of its gamma-carboxylic acid (Gla) domain, FXGL, was assayed in parallel experiments. As expected from previous studies (reviewed in [24]), we found that hexon binding to HS was enhanced in the presence of FX, but not with FXGL (**Fig. 1 A**), and this enhancing effect occurred in a FX dose-dependent manner (not shown). The binding of HAdV5wt virions to immobilized HS with and without FX was also assessed by SPR, using various stoichiometric ratios of FX per hexon trimeric capsomere, as determined from the number of virus particles present in the samples. FX enhanced the binding of HAdV5wt virions to HS in a dose-dependent manner (**Fig. 1 B**). As for isolated hexon protein, the Gla domainless FXGL showed no significant enhancement of the binding of HAdV5wt virion to HS (not shown). Of note, a weak signal of binding was observed with control samples of FX alone (**Fig. 1 A**), used in amounts equivalent to its average physiological concentration in human adult serum (8 µg/ml), referred to as the maximum FX concentration (FX_max_). This excluded the possibility that the signal of binding to HS observed with FX∶hexon or FX∶vector complexes were due to the binding of free FX.

Two mutants of HAdV5 were then analyzed, HAdV5F^TTAT^, mutated in the KKTK motif of the fiber shaft, and HAdV5Pb^EGD^, mutated in the RGD motif of the penton base. The KKTK tetrapeptide had been identified as a putative HSPG-binding motif [45], [46], [50], a function which is debatable [51]. FX used at 8 µg/ml (FX_max_) was found to enhance the binding of the two mutants to HS to equivalent levels (**Fig. 1 C**). As for HAdV5wt, no binding enhancement was observed with the Gla domainless version FXGL, indicating that the FX bridge between HAdV5wt hexon and HS required the integrity of its Gla domain. This confirmed previous reports which showed that the Gla domain of FX interacts with the hexon capsomere [26], [33], whilst basic residues Arg240, Lys236, Lys169, Arg165, Lys96, Arg93, and Arg125 in the exosite of FX interact with HSPG [52]. Our results also indicated that the mutations in HAdV5F^TTAT^ and HAdV5Pb^EGD^ had no effect on the FX-mediated binding function of HAdV5wt hexon to HS.

### Requirement of HSPG expression at the cell surface for FX-mediated enhancement of cell transduction by HAdV5wt vector

**Figure 2:**
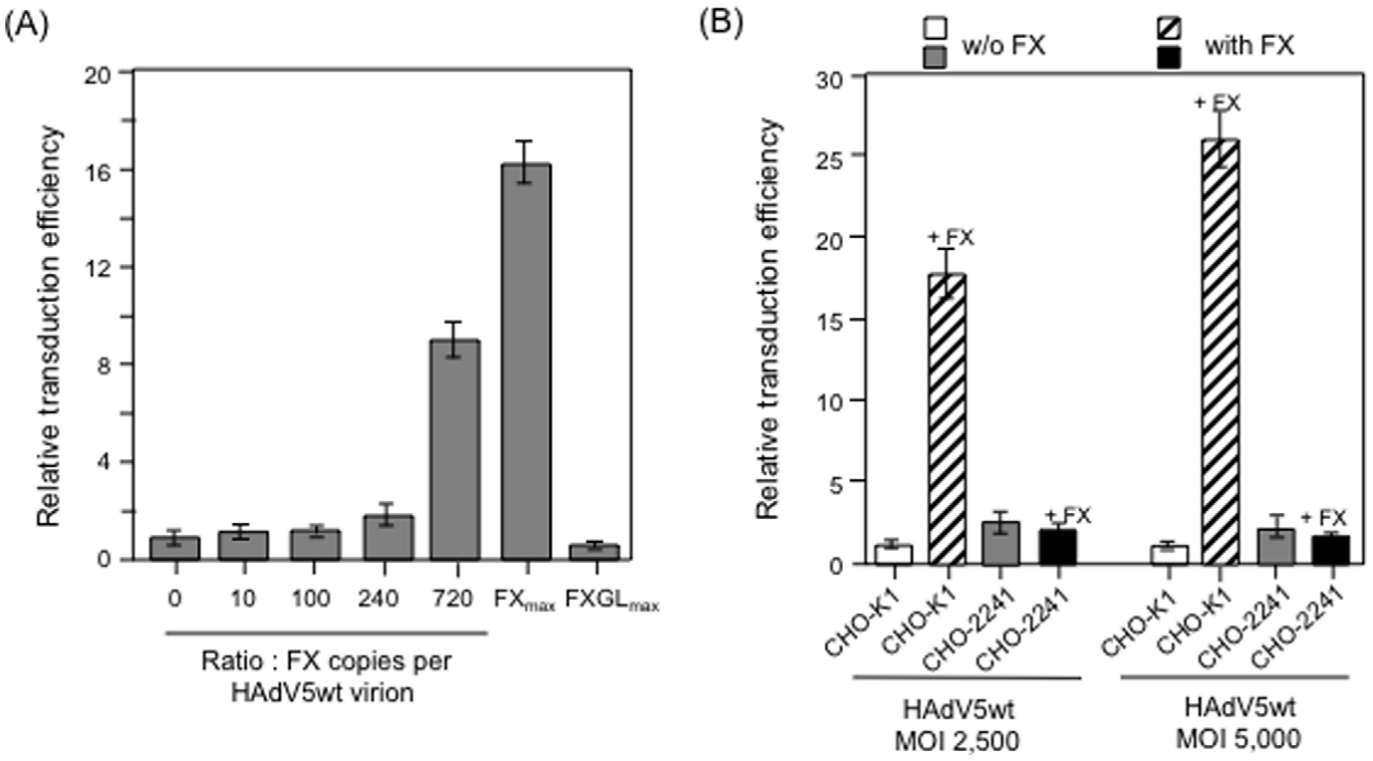
Cell transduction of CAR- and CD46-negative CHO cells by HAdV5wt in the absence of presence of FX. (**A**), Dose-response effect of FX on cell transduction. CHO-K1 cells were transduced by GFP-expressing HAdV5wt vector in the presence of increasing concentration of FX. Both FX_max_ and FXGL_max_ corresponded to 8 mg/ml. Results were expressed as relative transduction efficiency (RTE). Transduction efficiency, in arbitrary units (AU), was given using the formula∶TE = (percentage of GFP-positive cells)×(MFI). The RTE was calculated using the formula∶RTE = (TE with FX)∶(TE without FX), with the 1-value attributed to TE in the absence of FX. (**B**), CHO-K1 (double CAR- and CD46-negative cells) and CHO-2241 (triple CAR- , CD46-, and HSPG-negative cells) were transduced by HAdV5wt vector at MOI 2,500 (left half of the bar graph) or MOI 5,000 (right half of the bar graph) in the absence or presence of FX (8 µg/ml). Results were expressed as RTE, with the 1-value attributed to the TE of CHO-K1 in the absence of FX.

We next assayed the transduction efficiency of HAdV5wt vector in the presence or absence of FX or FXGL in a CAR-negative cellular model, using CHO cells, which express HSPG at their surface (control CHO-K1), or CHO-2241, which are deficient in HSPG expression. The FX concentrations in the viral inoculum ranged from 0 to 8 µg/ml (FX_max_). In HSPG-positive CHO-K1 cells, FX, but not the Gla domainless FXGL, enhanced the transduction efficiency (TE) of HAdV5wt in a dose-dependent manner (**Fig. 2 A**). Of note, the maximum enhancement of transduction was not reached with a concentration of FX in the medium corresponding to a ratio of 720 copies of FX per virion (*i.e.* with 3 FX per trimeric hexon capsomere), but with the highest FX concentration (FX_max_; **Fig. 2 A**), a result consistent with sensorgrams shown in Fig. 1B. At FX_max_, the increase of TE was 18-fold at 2,500 vp/cell, and 26-fold at 5,000 vp/cell (**Fig. 2 B**). No detectable effect of FX on TE levels was observed in HSPG-negative CHO-2241 cells (**Fig. 2 B**). This demonstrated that the surface expression of HSPG molecules was indispensable for the FX-mediated enhancing effect on the HAdV5wt cell binding and transduction, confirming previous studies [24], [37].

### Absence of requirement of fiber KKTK motif and penton base RGD motif for FX- and HSPG-mediated enhancement of cell transduction by HAdV5 vectors

**Figure 3:**
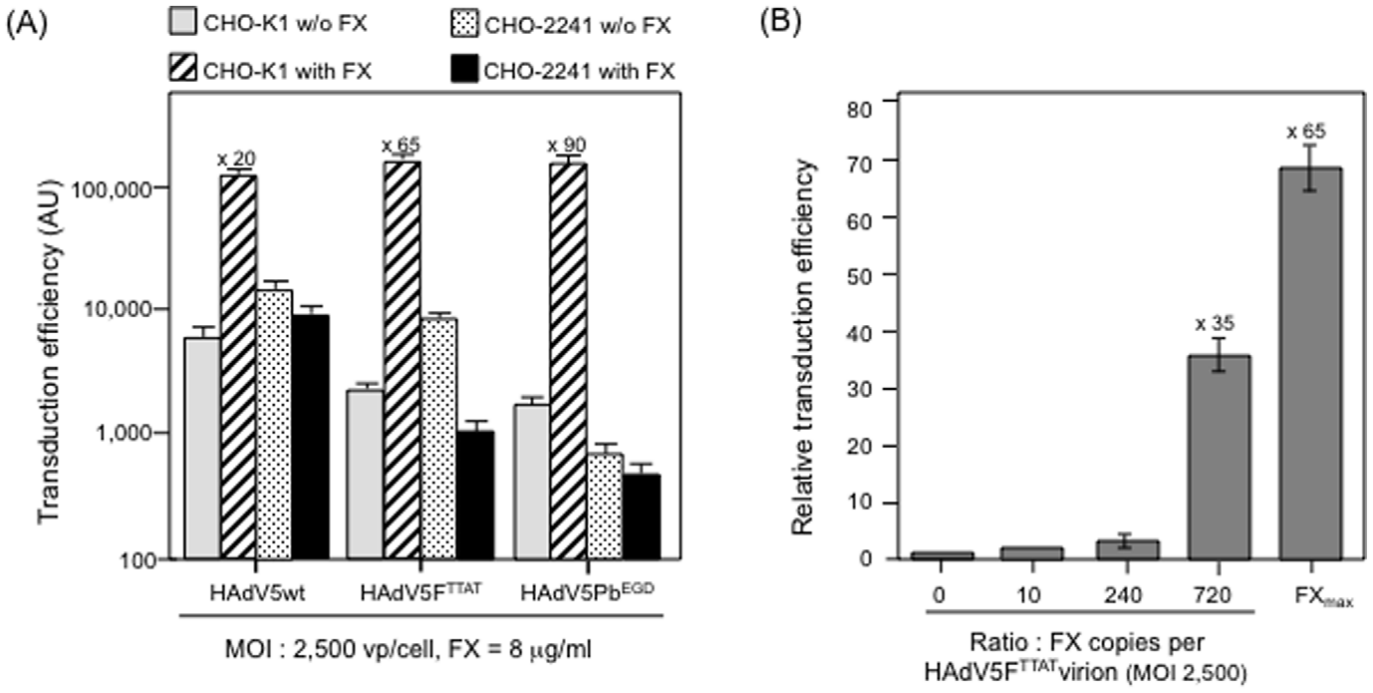
Transduction of CHO-K1 or CHO-2241 cells by GFP-expressing, fiber mutants of HAdV5-based vectors in the absence (w/o) or presence of (with) FX (8 µg/ml). (**A**), HAdV5wt, mutants HAdV5F^TTAT^ and HAdV5Pb^EGD^ and serotype 35 fiber-pseudotyped HAdV5F35 were all used at MOI 2,500, and transduction efficiency were expressed as arbitrary units (AU), as described in the legend to Fig. 2. (**B**), Dose-response effect of FX on cell transduction by HAdV5F^TTAT^ mutant. CHO-K1 cells were transduced by GFP-expressing HAdV5F^TTAT^ mutant vector in the presence of increasing concentration of FX (FX_max_ = 8 µg/ml). Results were expressed as relative transduction efficiency (RTE; refer to the legend to Fig. 2).

It was shown that mutation of the putative HSPG binding site in the HAdV5wt shaft (^91^KKTK^94^) interfered negatively with the cellular trafficking of the virions to the nucleus [45], [51]. We therefore evaluated the influence of FX on the capacity of transducing CHO-K1 and CHO-2241 cells by the mutant vector HAdV5F^TTAT^. In the absence of FX, HAdV5F^TTAT^ transduced CHO-K1 cells with a significantly lower TE, compared to HAdV5wt used at the same MOI (2,500 vp/cell ; **Fig. 3 A**). In the presence of increasing doses of FX, we observed a progressive augmentation of the TE, with a 35-fold enhancement for the FX∶virion ratio of 3∶1 for (*viz*. 720 copies of FX per 240 hexon capsomeres), and 65-fold for FX_max_ (**Fig. 3 B**). No enhancement, but instead a slight decreasing effect of FX on TE was observed in CHO-2241 (**Fig. 3 A**). This indicated that FX was able to rescue the loss of infectivity due to the KKTK-to-TTAT mutation in the fiber shaft, provided that HSPG molecules were present at the cell surface. This also indicated that the putative HSPG-binding motif KKTK was dispensable for the FX-mediated bridging of HAdV5wt virion to surface HSPG molecules, confirming the major role of hexon as the ligand of FX [24]. It could not be excluded however that other fiber interactions, besides the assumed interaction with HSPG, might be affected as a consequence of the TTAT mutation, although the TTAT mutant fibers folded as trimers and were incorporated at wild-type levels in the adenoviral capsid (data not shown).

Human adenovirus serotype 35 (HAdV35) utilizes CD46 and α_v_-integrins as primary and secondary receptors, respectively [41], [53]. We next investigated the possible influence of RGD-dependent integrins on the FX+HSPG-mediated enhancing effect on transduction by HAdV5 vectors. To this aim, the HAdV5Pb^EGD^ penton base mutant was used in transduction assays of CHO-K1 and CHO-2241 cells. In the absence of FX, HAdV5Pb^EGD^ showed a lower TE of both CHO-K1 and CHO-2241 cells, compared to HAdV5wt used at the same MOI (**Fig. 3 A**). In the presence of FX_max_, we observed an enhancement of transduction of CHO-K1 cells, at levels similar to those of HAdV5wt or HAdV5F^TTAT^ (50- to 100-fold), whereas no effect was detectable in CHO-2241 (**Fig. 3 A**). This showed that FX was able to rescue the negative effect of the penton base RGD-to-EGD mutation in HSPG-expressing CHO-KI cells, but not in HSPG-negative CHO cells. Similar to the HAdV5F^TTAT^ fiber shaft mutant, this result implied that the penton base RGD motifs and the integrins played no significant role in the FX+HSPG-mediated enhancement of cell transduction using HAdV5 vectors.

Interestingly, CHO-2241 seemed to be slightly more permissive to HAdV5wt compared to CHO-K1 infected at the same MOI in the absence of FX, which could suggest a higher accessiblity of alternative virus attachment receptor(s) other than HSPG for the primary binding of HAdV5wt to CHO-2241 cells, e.g. integrins [12], [54], [55]. The possibility of integrins acting as alternative attachment receptor of Ad5 to CHO cells in the absence of CAR and HSPG were envisaged, based on the results obtained with our RGD-mutant vector : HAdV5Pb^EGD^ transduced CHO-2241 cells in the absence of FX_max_ with a 10-fold lower efficiency, compared to HAdV5wt (**Fig. 3 A**). However, since CHO cells lack ß-integrins and fail to express α_V_ß3/5 integrin heterodimers at their surface, other types of RGD-interactors/ligands might be responsible for the low levels of HAdV5Pb^EGD^-mediated transduction.

### Paradoxical behavior of serotype 35 fiber-pseudotyped HAdV5F35 vector in the context of FX and HSPG

Fiber swapping between adenovirus serotypes has been widely used as a rational strategy to *(i)* explore the various functions associated with fibers in fundamental virology [56]–[58], *(ii)* to allow fiber-pseudotyped vectors to evade prexisting neutralizing antibodies [59], [60], or *(iii)* to ablate the natural tropism of the virus and confer a novel transductional retargeting capacity to the pseudotyped vectors [24], [27], [34], [37], [61]–[67]. Novel and sometimes unexpected properties, *e.g.* nonnative entry pathway and/or aberrant cellular trafficking, have been observed with such chimeric adenoviruses. This was the case for the serotype 35 fiber-pseudotyped HAdV5F35 vector [68], or for HAdV5F2/BAdV4, which carried bovine-human chimeric fibers [39].

**Figure 4:**
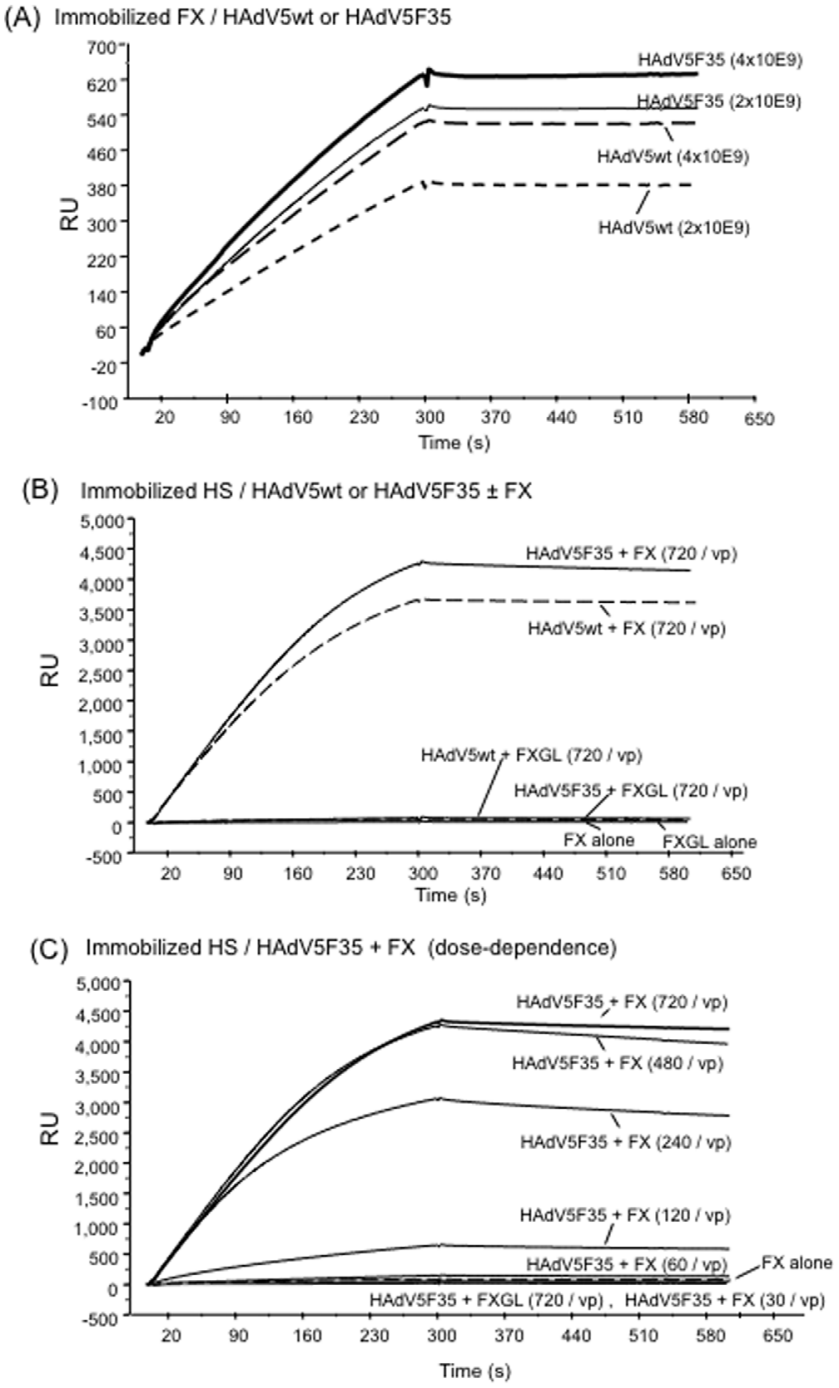
SPR analysis of the *in vitro* binding of chimeric HAdV5F35 vector to (A) surface-immobilized FX, or (B, C) immobilized HS with or without FX or FXGL. (**A**), Representative sensorgrams for HAdV5wt vector (discontinuous lines) injected at 2×10^9^ and 4×10^9^ vp/ml, or for HAdV5F35 injected at the same doses (solid lines). (**B**), Comparison of binding to HS of HAdV5wt and HAdV5F35 vector particles (2×10^9^ vp/ml) in the presence of FX or FXGL at 720 copies per vector particle. Controls shown are FX and FXGL alone. For better clarity, the sensorgrams for virions alone, which superimposed those of FX and FXGL, are not shown (refer to Fig. 1C). (**C**), Dose-response effect of FX on HAdV5F35 binding to immobilized HS. Note that a detectable signal was observed for 120 copies of FX per virion, and reached the maximal value for 480 copies/vp.

A phylogenetic study of HAdV fiber shaft has revealed the absence of the putative heparan sulfate (HS)-binding site (KKTK motif) in all HAdVs other than species C [69]. The serotype 35 fibers carried by the chimeric HAdV5F35 vector lacked the KKTK motif, and species B HAdV35 has been shown to interact with cellular HSPG via capsid protein domains other than the fiber knob [42]. We therefore wanted to determine the capacity of our chimeric HAdV5F35 vector to bind to HS with or without a FX bridge. Firstly, we analyzed the binding of HAdV5F35 to immobilized FX in SPR assays, and found that HAdV5F35 bound to FX with a higher response compared to HAdV5wt used at the same particle input (**Fig. 4 A**). We also observed a higher FX-mediated binding of HAdV5F35 to surface-immobilized HS, compared to HAdV5wt (**Fig. 4 B**). No effect was observed with FXGL (**Fig. 4 B**). In dose-dependent binding assays, maximum binding was observed with 480 copies of FX per HAdV5F35 virion, *i.e.* a stoichiometric ratio of 2 FX molecules per hexon capsomere, with no further significant increase obtained using 3 copies of FX per hexon (720 FX copies per virion; **Fig. 4 C**). This differed from the sensorgrams of FX-mediated binding of HAdV5wt to immobilized HS, which was not maximal with 2 copies of FX per hexon, and still increased in the range 2 to 10 copies of FX per hexon (refer to **Fig. 1 C**). This difference could be due to the relatively higher accessibility of hexon capsomeres to FX in short fiber-carrying HAdV5F35 vector, compared to the long fiber-carrying vector HAdV5Fwt.

**Figure 5:**
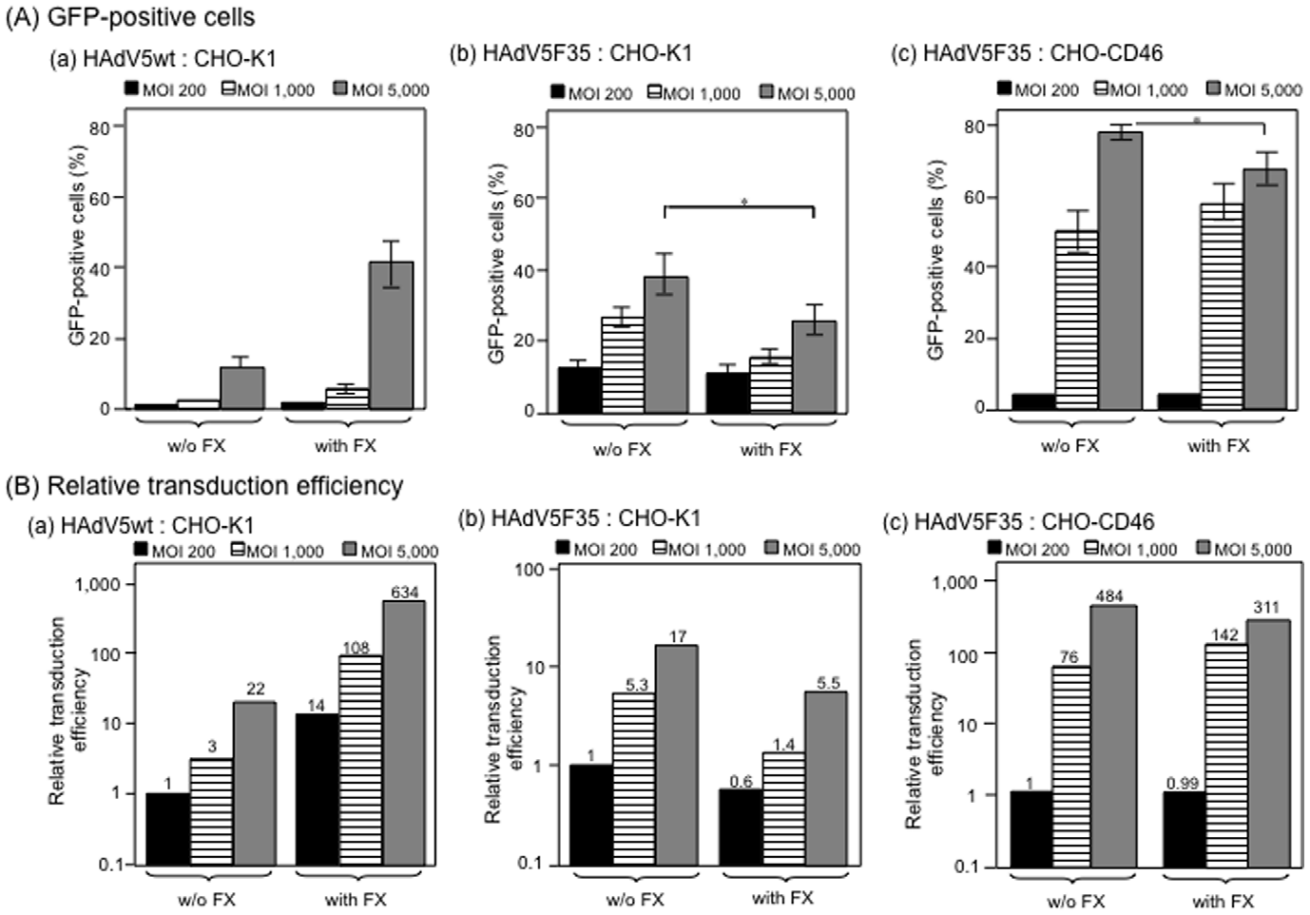
Comparison of transduction efficiency of (a, b) CHO-K1 cells or (c) CHO-CD46 by (a) HAdV5wt, and (b, c) chimeric HAdV5F35 vectors at different MOI (200, 1,000 or 5,000 vp/cell) in the absence (w/o) or presence of (with) FX (8 µg/ml). Results were expressed as (**A**) the percentage of GFP-positive cells, or (**B**) relative transduction efficiency (RTE; refer to the legend to Fig. 2). In B, the number on top of each bar corresponded to the fold increase in RTE, with the 1-value attributed to the TE of CHO-K1 or CHO-CD46 cells transduced by HAdV5F35 at MOI 200. Note that RTE of CHO-K1 cells with HAdV5F35 was lower in the presence of FX than in the absence of FX, at all MOI tested.

The influence of FX-mediated bridging of HAdV5F35 to cellular HSPG on the transduction of CHO-K1 cell was then tested with increasing MOI, from 200 to 5,000 vp/cell. HAdV5wt was used at the same MOI for comparison and as control. As expected, the transduction efficiency with HAdV5wt vector was significantly higher when FX was added to the virus inoculum, from 14-fold to 30-fold higher at MOI 200 and 5,000 vp/cell, respectively (**Fig. 5, A and B** **, panels a**). With HAdV5F35 however, no enhancing effect on transduction of CHO-K1 cells was observed in the presence of FX, compared to the vector alone, but instead a 3- to 4-fold decreasing effect at high MOI (**Fig. 5, A and B** **, panels b**). Likewise, in control experiments using HAdV5F35 and permissive CHO-CD46 cells, no increasing effect of FX on cell transduction was observed, but a decreasing effect at high vector doses (**Fig. 5, A and B** **, panels c**). A similar phenomenon has previously been reported, and attributed to a blockage in postinternalization step(s) of chimeric vectors carrying serotype 35 fibers [37]. The fact that FX-HAdV5F35 complexes were less efficient than FX-HAdV5wt complexes in cell transduction was paradoxical, considering *(i)* that both HAdV5wt and HAdV5F35 carried the same hexon capsomeres of serotype 5, *(ii)* which had the same ligand, FX, and *(iii)* that the FX-HAdV5F35 complexes were capable of binding to surface-immobilized HS and to cellular HSPG with an apparent higher affinity than that of the FX-HAdV5wt complexes.

### Cell attachment and internalization of FX-HAdV5F35 complex in HSPG-expressing CHO cells

The following experiments were designed to explain the 2-log difference between FX-HAdV5wt and FX-HAdV5F35 complexes in the transduction of CHO-K1 cells. We investigated the (i) cell attachment, (ii) cellular uptake (endocytosis and internalization), and (iii) intracellular trafficking of FX-HAdV5F35, in comparison with FX-HAdV5wt complexes, to determine which step(s) was blocked or altered in the infection pathway. The rationale for using CHO-K1 cells as target cells, was that they express neither CAR nor CD46, and thus made possible the analysis of the cell entry pathway of the HAdV5wt and HAdV5F35 vectors mediated by their FX-bridging to cellular HSPG, while avoiding any bias due to adenovirus serotype-specific receptors.

#### (i) Cell attachment

**Figure 6:**
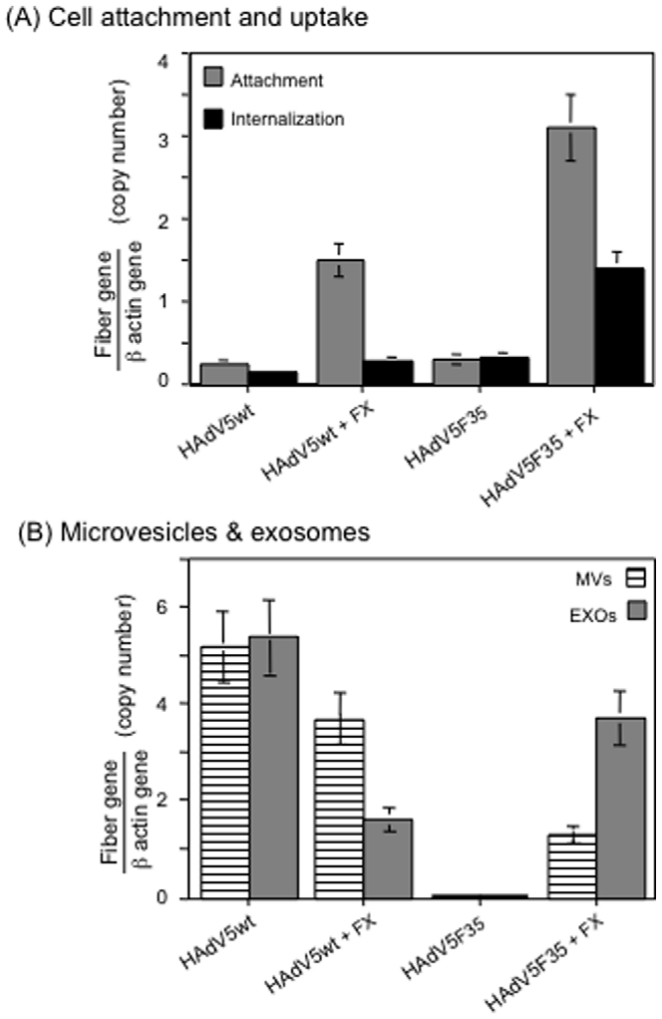
Cellular uptake and extracellular release of HAdV5wt and HAdV5F35 vectors by CHO-K1 cells. (**A**), Cell attachment of vector (MOI 5,000) was performed at 4°C for 1 h, and cellular internalization at 37°C for 1 h, respectively, with or without FX (8 µg/ml), as indicated on the *x*-axis. The number of viral genome copies was determined by qPCR of the fiber gene, normalized to the ß-actin gene. (**B**), Extracellular vectors associated with microvesicles (MVs) or exosomes (EXOs) recovered from the extracellular medium at 72 h post transduction, were determined as above.

Samples of HAdV5wt and HAdV5F35 suspension were mixed with FX at a final concentration of 8 µg/ml, and the mixture added to CHO-K1 cell monolayers at a MOI of 5,000 vp/cell. Incubation was carried out for 1 h at 4°C, which allowed for virus-cell attachment but not virus entry or endocytosis [70]. After extensive rinsing to remove unattached virus, cells were harvested and cell-bound virus determined by quantitative PCR analysis (qPCR) of viral genomes after DNA extraction, based on the fiber gene copy number normalized to the ß-actin gene. FX significantly increased the amounts of cell-bound virions, by a factor of 5 to 6 for HAdV5wt, and by a factor of 10 for HAdV5F35 (**Fig. 6 A**). This confirmed the apparent higher affinity of FX for HAdV5F35 as observed *in vitro*, compared to HAdV5wt (refer to Fig. 4). However, this was in apparent contradiction with the lower transduction efficiency of the FX-HAdV5F35 complex, compared to FX-HAdV5wt (refer to Fig. 5), and excluded the cell attachment as the limiting step for FX-HAdV5F35-mediated transduction.

#### (ii) Cellular uptake

The subsequent step of endocytosis was then investigated. After an incubation period of 1 h at 4°C with FX-vector complexes at MOI 5,000, as above, samples of CHO-K1 cells were transferred to 37°C and harvested after 1 h. After a brief trypsin treatment, to remove vector particles remaining bound to the plasma membrane [71], cell samples were subjected to DNA extraction and viral genomes determined by qPCR, as above. In the absence of FX, the intracellular content was not significantly different between HAdV5wt and HAdV5F35. In the presence of FX, there was a 2-fold increase in HAdV5wt uptake, and a 4-fold increase for HAdV5F35 (**Fig. 6 A**). This suggested that the mechanism responsible for the lower cell transduction by the FX-HAdV5F35 complex did not involve the endocytic step of the vector.

#### (iii) Extracellular release

The previous results incited us to explore the exocytic pathway of cell-internalized vector particles, i.e. the possibility that significant amounts of HAdV5F35 particles might be released in the extracellular medium, either by shedding of vector-containing microvesicles (MVs) budding from the plasma membrane, or by exocytosis, i.e. the release of exosomes (EXOs) segregated within the lumen of multivesicular bodies (MVBs) (reviewed in [72]). CHO-K1 cell culture supernatants were harvested at 2 h and 24 h post incubation with of HAdV5wt or HAdV5F35 at MOI 5,000, with or without FX. The extracellular MVs and EXOs from the culture supernatants were separated by sequential and differential ultracentrifugation. After DNA extraction, the amount of virus particles in each microparticle population was determined by qPCR quantification of the viral genomes. In the absence of FX, HAdV5wt genomes were recovered in significant amounts in MVs and EXOs, and these amounts decreased in both types of microparticles when transduction was performed in the presence of FX (**Fig. 6 B**). Interestingly, the profile was different for HAdV5F35, with and without FX: (i) HAdV5F35 genomes were undetectable in either microparticle population in the absence of FX, but became detectable in the presence of FX; (ii) HAdV5F35 was recovered in higher amounts in EXOs, compared to MVs (2-fold; **Fig. 6 B**). This pattern suggested that the intracellular trafficking was likely responsible for the low efficiency of FX-HSPG-mediated transduction of CHO-K1 cells by HAdV5F35, and the next experiments were designed to determine which subcellular compartment(s) were possibly implicated.

### Intracellular trafficking and compartmentalization of FX-HAdV5F35 complexes in HSPG-expressing CHO cells

#### (i) Live cell imaging

**Figure 7:**
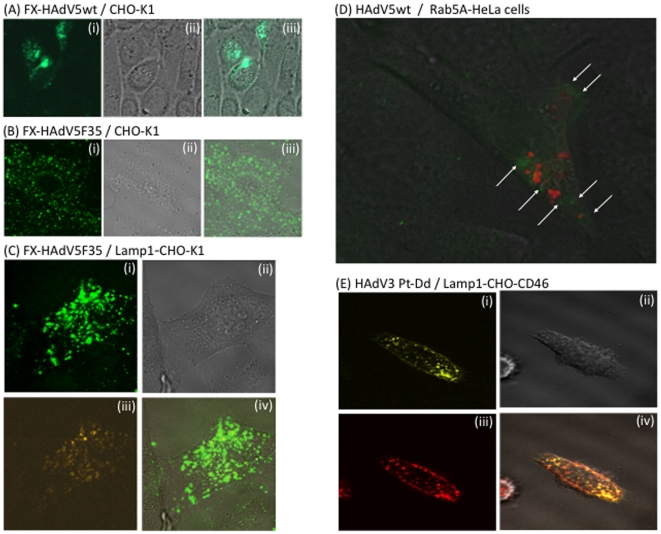
Confocal microscopy of live cells transduced by adenoviral vector particles or capsid components (penton dodecamers). (**A–C**), Confocal microscopy of live cells (CHO-K1) transduced by Alexa-488-labeled adenoviral vectors, used at 10,000 vp/cell and complexed with FX (8 µg/ml). (**A**) HAdV5wt, 30 min pi; (**B, C**) HAdV5F35, 3 h pi. (i), Green channel image; (ii), phase contrast; (iii), merge of (i) and (ii). In (**C**), CHO-K1 cells were transduced by recombinant baculoviral vector expressing RFP-tagged, late endosome marker Lamp1 protein, 24 h before incubation with HAdV5F35 vector. (i), Green channel image; (ii), phase contrast; (iii) orange channel; (iv), merge of (i) and (iii). (**D**) Live HeLa cells transduced by recombinant baculovirus expressing RFP-tagged, early endosome marker Rab5A protein, were incubated 24 h later with Alexa-488-labeled HAdV5wt particles without FX, at 10,000 vp/cell and 37°C. Picture shown was taken at 20 min after incubation with HAdV5wt. Note that most of the virus signal is weak and diffuse, but some green fluorescent dots are visible within the cytoplasm (white arrows). (**E**), Live CHO-CD46 cells transduced by recombinant baculovirus expressing RFP-tagged, late endosome marker Lamp1 protein, were incubated 24 h later with Cy5-labeled HAdV3 penton dodecahedrons (Pt-Dd) at 37°C. Picture shown was taken at 60 min after incubation with Pt-Dd. (i), Cy5 channel; (ii), phase contrast image; (iii) RFP channel; (iv), merge of (i) and (iii).

The capsids of HAdV5wt and HAdV5F35 vectors were chemically labeled with Alexa-488, and mixed with FX, before incubation with CHO-K1 cells at high vector multiplicity (10,000 vp/cell). Intracellular virions were tracked *in situ* in live cells by time-lapse microscopy at early times of infection (0 to 4 h pi). As early as 20–30 min pi, most of the HAdV5wt signal was found in the vicinity of or within the nucleus (**Fig. 7 A**). This observation was consistent with the well-described rapid process of endocytosis, endosomal escape and intracellular trafficking of HAdV5wt virions [22], [73], and suggested that FX had no significant effect on the internalized HAdV5wt particles and the kinetics of their intracellular transit. This implied that FX acted at earlier steps, an hypothesis consistent with the role of molecular bridge between viral hexon capsomeres and cell surface HSPG played by FX. The fluorescence pattern of HAdV5F35 vector was however significantly different. At 30 min pi, no fluorescent signal was observed in the nucleus, instead multiple fluorescent dots were visible in the cytoplasm, and most of the fluorescence remained cytoplasmic until 3 h pi (**Fig. 7 B**). This suggested that HAdV5F35 particles were delayed in terms of intracellular trafficking, compared to HAdV5.

#### (ii) Retention of HAdV5F35 particles in the late endosomal compartment

To determine the nature of the subcellular compartment in which HAdV5F35 was retained, CHO-K1 cells were transduced by baculovirus vectors expressing fluorescent markers designed for live-imaging of different cellular organelles or compartments. We found that the green fluorescent signal of HAdV5F35 colocalized with red fluorescent Lamp1 protein [74], [75], a marker of the lysosomal/late endosomal compartment (**Fig. 7 C**). This result suggested that the retarded trafficking to the nucleus of HAdV5F35 particles was due to their segregation into the late endosomal compartment.

#### (iii) Evaluation of the baculovirus-mediated labeling of cellular organelles and compartments for the study of adenoviral vector pathway in living cells

To verify the validity of our observation of late endosomal compartmentalization of HAdV5F35 particles, HeLa, HEK-293, CHO-K1 or CHO-CD46 cells were transduced with recombinant baculoviruses expressing Lamp1-RFP, as above, or Rab5A-RFP [76], a marker of the early endosomes, 24 h prior to incubation with Alexa-488-labeled HAdV5wt particles or Cy5-labeled HAdV3 penton dodecahedrons (Pt-Dd). No image of colocalisation of Alexa-488 labeled HAdV5wt with RFP-labeled early endosomes could be captured, even after short incubation period: as early as after 10–15 min incubation with living cells at 37°C, green fluorescent dots of HAdV5wt were already found within the cytoplasm, outside of the red fluorescent endosomal compartment (**Fig. 7 D**). This confirmed the scenario described by Greber et al., which showed that incoming HAdV5wt particles escaped very rapidly from early endosomes [73]. By contrast, Cy5-labeled Pt-Dd localized in the late endosomal compartment of HeLa or CHO cells after incubation at 37°C for 1 h (**Fig. 7 E**), as expected for the HAdV3 pathway [40], [77]–[79]. The usefulness of baculovirus-mediated labeling of specific organelles for tracking adenovirus particles or adenoviral components within living cells was therefore validated using our in-house model of HAdV3 Pt-Dd.

### Electron microscopy (EM) of cell-internalized HAdV5wt and HAdV5F35 particles in the presence of FX

**Figure 8:**
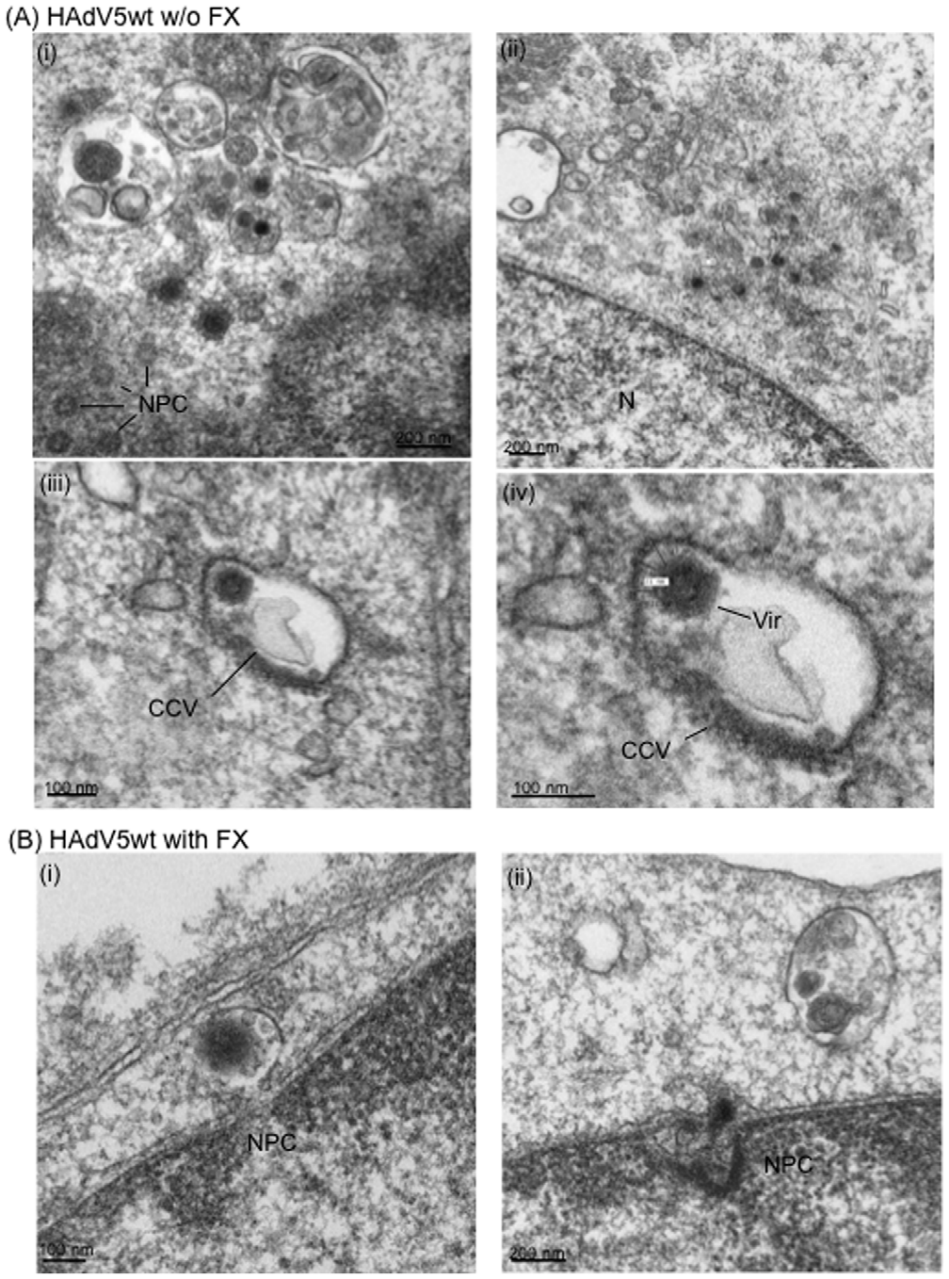
Electron microscopy of CHO-K1 cells incubated with HAdV5wt at 10,000 vp/cell, (A) in the absence (w/o), or (B) presence of FX (8 µg/ml) for 2 h at 37°C. (**A**), (i) and (ii): General views of cell sections showing (i) intravesicular and (ii) cytoplasmic vector particles. In (iii) and (iv), a vector particle (Vir) is seen within a clathrin-coated vesicle (CCV); (iv), enlargement of the CCV shown in (iii), with measurements of the space between the vector particle and the inner leaflet of the vesicular membrane. N, nucleus; NPC, nuclear pore complexes viewed in a tangential section. (**B**), (i): vector particle within an endocytic vesicle in the vicinity of a nuclear pore; (ii), viral core seen in the process of traverse of the nuclear pore.

To further explore the cellular localization of internalized vector particles in the presence of FX, CHO-K1 cells were incubated with HAdV5wt or HAdV5F35 vector (MOI 10,000) alone or complexed with FX (8 µg/ml). Cells were harvested at 2 h pi at 37°C, and processed for EM. As expected for CAR-negative cells transduced in the absence of FX, rare HAdV5wt virions were observed within the cells, in vesicles (**Fig. 8 A, i**) or free in the cytoplasm (**Fig. 8 A, ii**). Occasionally, HAdV5wt virions were seen attached to the invaginated plasma membrane forming clathrin-coated vesicles: in such cases, the average distance of the capsid to the plasma membrane was found to be 25±4 nm (m ± SEM), a value consistent with the length of serotype 5 fiber (**Fig. 8 A, iii and iv**). This suggested that in the absence of CAR and FX, HAdV5wt bound directly to components of the CHO-K1 plasma membrane acting as alternative receptors. In the presence of FX however, HAdV5wt found in vesicles seemed to be associated with electron lucent material (**Fig. 8 B, i**). Very rare HAdV5wt particles were found within the cells at 2 h pi, likely due to their rapid transit to the nucleus and traverse of the nuclear pore (**Fig. 8 B, ii**).

**Figure 9:**
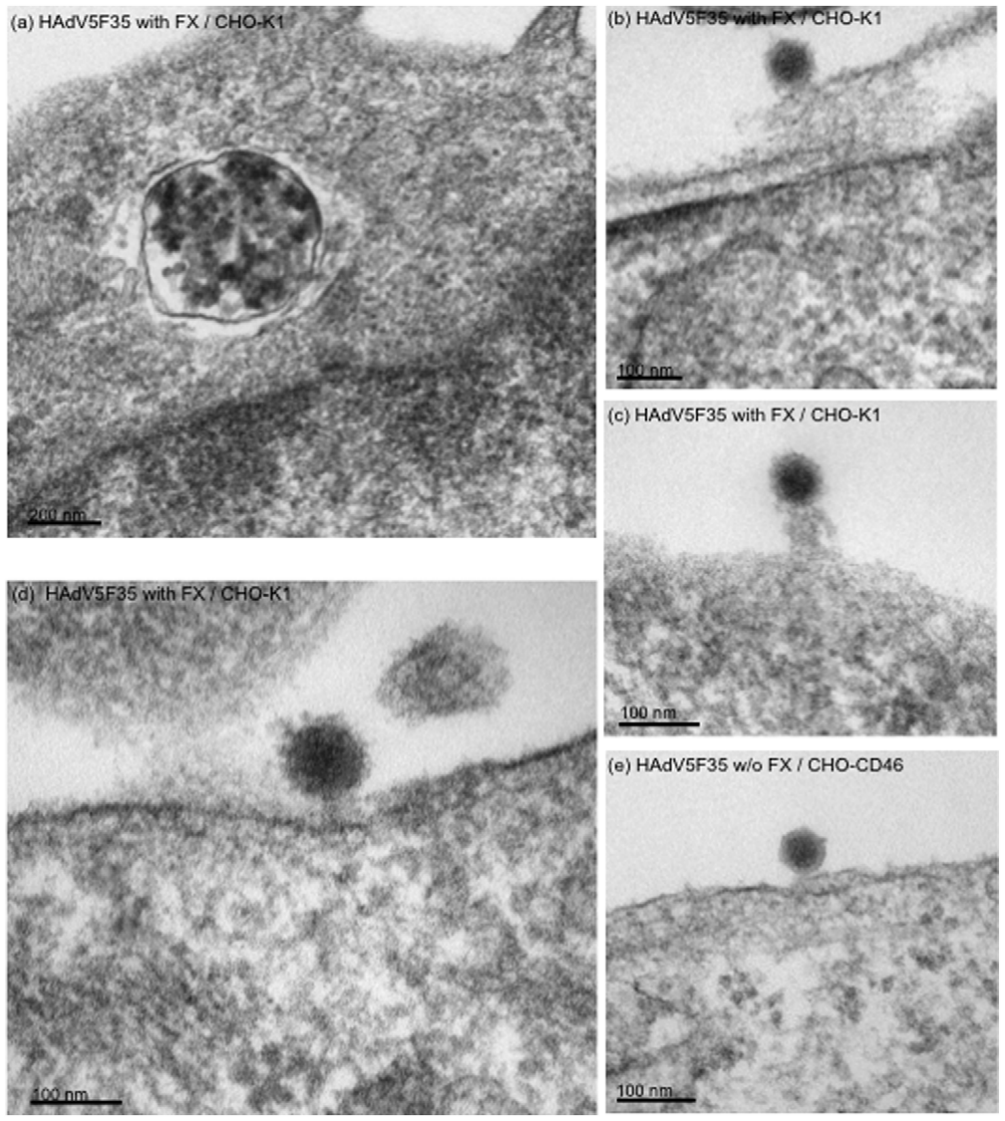
Electron microscopy of CHO-K1 cells (a–d) incubated with HAdV5F35 at 10,000 vp/cell in the presence of FX (8 µg/ml), and harvested after 2 h at 37°C. (**a**), Representative CHO-K1 cell section showing a cytoplasmic vesicle containing abundant electron dense material. (**b–d**), Cell surface-bound HAdV5F35 particles. (**e**), CHO-CD46 cells incubated with HAdV5F35 in the absence of FX (w/o FX). Note the difference in size and sharpness of the viral contour between HAdV5F35 particles seen in (e) and in (b–d).

The pattern was different for CHO-K1 cells incubated with FX-HAdV5F35 complex for 2 h at 37°C. No particle with the regular shape of adenovirions was observed in any of the cellular compartments, but each cell section showed large vesicles containing abundant, electron dense material (**Fig. 9 a**). This pattern was consistent with the results of confocal microscopy showing the accumulation of FX-HAdV5F35 complexes in lysosomes (refer to Fig. 7). Interestingly at the cell surface, HAdV5F35 particles were frequently seen connected to the plasma membrane *via* a bridge consisting of filamentous material (**Fig. 9 b–d**). The length of these bridges varied from 80 to 140 nm, with an average at 105 nm, a value compatible with the thickness of the fibrilllous glycocalyx coat (77 to 201 nm; [80]). By contrast, in control CHO-CD46 cells incubated with HAdV5F35 vector in the absence of FX, cell-bound particles were seen at a distance of 13±2 nm from the plasma membrane outer leaflet (**Fig. 9 e**), a value compatible with the short-shafted serotype 35 fiber bound to its CD46 receptor. Enlargements of cell-bound HAdV5F35 particles showed fuzzy material decorating the viral capsid (**Fig. 9 d**), conferring the whole complex a diameter of 107±6 nm, instead of 80–85 nm for free virions (**Fig. 9**: compare the sharp contour of the control HAdV5F35 virion in panel **e** to the blurred contour of FX-HAdV5F35 complex in panel **d**). We hypothesize that the filaments emanating from the cell which bridged the vector to the cell surface represented HSPG, components of the cell glycocalyx [80], whereas the fuzzy material which coated the capsid corresponded to FX molecules bound to hexon.

### Cellular uptake and retention of FX with or without adenoviral particles

**Figure 10:**
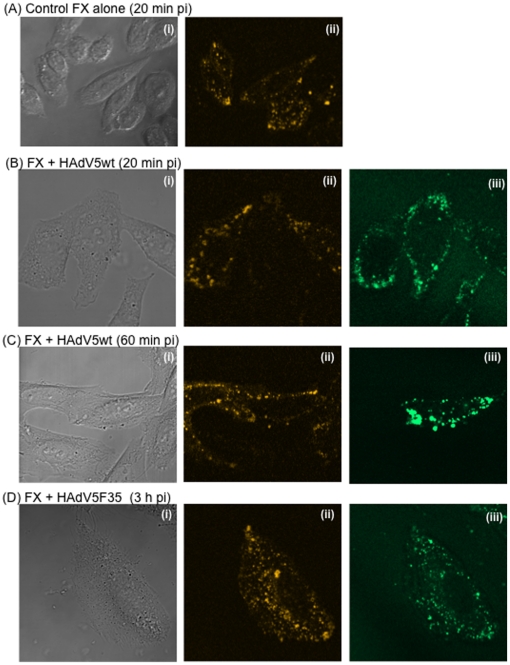
Confocal microscopy of live cells (CHO-K1) incubated with (A) Alexa-555-labeled FX alone (8 µg/ml), or (B, C) in complex with Alexa-488-labeled HAdV5wt, or (D) in complex with Alexa-488-labeled HAdV5F35, both vectors used at 10,000 vp/cell. Images were taken at 10-min intervals, until 3 h post incubation (pi). (i), phase contrast image; (ii) orange channel; (iii) green channel.

Our observation that FX enhanced the vector-cell binding for both HAdV5wt and HAdV5F35, but failed to augment the FX-HAdV5F35-mediated cell transduction, raised the question of the fate of FX after the cell attachment of the FX-vector complex: was FX coendocytosed with HAdV5wt or HAdV5F35, or did it remain outside of the cell? To address this issue, Alexa-555-labeled FX was preincubated with Alexa-488-labeled vector particles, and the complex added to CHO-K1 cells. As control, Alexa-555-labeled FX was added to CHO-K1 cells alone, without preincubation with the vector. Both types of samples were followed by live cell imaging. We found that FX alone could bind to CHO-K1 cells and was rapidly endocytosed: intracellular FX was detected as early as 15–20 min post incubation (**Fig. 10 A**). No detectable cellular uptake of FX was observed in HSPG-deficient CHO-2241 cells (not shown). In CHO-K1 cells incubated with the double labeled FX-HAdV5wt complex, both fluorescent signals were observed within the cell at 15–20 min pi, and most signals colocalized in cytoplasmic dots and patches (**Fig. 10 B**). At later times pi (45–60 min), HAdV5wt particles were found to localize at the nuclear periphery or inside the nucleus, and less colocalization with FX was visible (**Fig. 10 C**). In CHO-K1 cells incubated with double labeled FX-HAdV5F35 complex, FX and virions remained colocalized within the cytoplasm until late times pi (3 h pi; **Fig. 10 D**). This confirmed the rapid intracellular transit of HAdV5wt, in contrast to the slow trafficking and vesicular retention of HAdV5F35.

**Figure 11:**
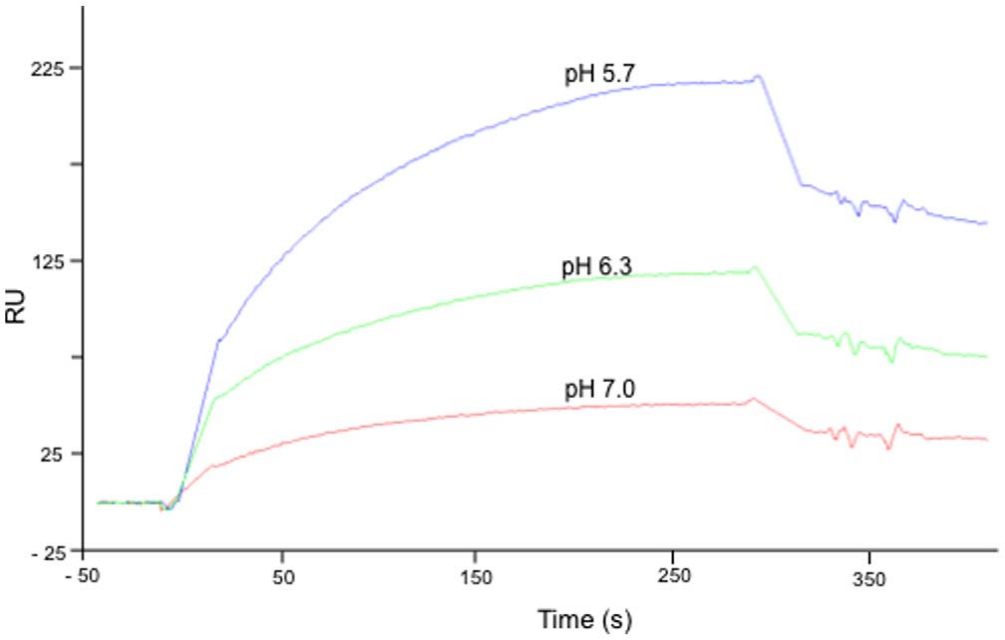
SPR analysis of the pH-dependence of FX∶hexon protein interaction. FX was immobilized on the sensorchip, and a hexon protein solution in 150 mM NaCl, 0.05 M sodium phosphate buffer of various pH values was injected into the flowcell.

We next investigated whether the vesicular retention or sequestration of HAdV5F35 could be explained by a higher stability of FX-HAdV5F35 complex in the acidic environment of the late endosomal compartment, compared to the higher pH of early endosomes, the endocytic compartment of HAdV5wt. This was determined indirectly, using SPR analysis of the apparent affinity between FX and serotype 5 hexon, the capsomeres which were in common between HAdV5F35 and HAdV5wt and the targets of FX. FX was covalently immobilized onto the biosensor chip, and hexon protein diluted in the same 0.05 M phosphate buffer but with the different Na_2_HPO4∶NaH_2_PO4 ratios required to obtain the desired pH values. The sensorgrams obtained showed that the profile of binding of serotype 5 hexon to FX was significantly higher at pH 5.7, compared to neutral pH, with an intermediate interaction observed at pH 6.3 (**Fig. 11**). This suggested that the FX-HAdV5F35 complex dissociated at slower rate in the acidic environment of late endosomal vesicles, compared to that of FX-HAdV5 complex in early endosomes. This vesicular sequestration would account for the lower transduction efficiency of (i) the FX-HAdV5F35 complex, compared to FX-HAdV5wt complex (refer to Fig. 5, panels a and b), and (ii) of the FX-HAdV5F35 complex, compared to the HAdV5F35 vector alone (refer to Fig. 5, panels b).

## Discussion

It was recently shown that HAdV5 interaction with human blood coagulation FX, which results in the formation of FX-HAdV5 complexes, is the major parameter responsible for the massive liver uptake of HAdV5 vector particles administered systemically. FX binds to the hexon capsomeres via its Gla domain, and subsequently interacts with cell surface-exposed HSPG molecules which are present in abundance at the surface of liver Kupffer cells [25]–[34]. This prompted several laboratories to engineer HAdV5-based vectors with hexon modifications designed to abolish the FX binding and reduce their hepatotropism (reviewed in [24]). However, the species B member HAdV35 [36], the serotype 35 fiber-pseudotyped vector HAdV5F35 [37], and the HAdV5/35 chimeric vector carrying serotype 35 fiber knobs [38] all showed a decreased hepatotropism, compared to HAdV5. Likewise HAdV5F2/BAdV4, another fiber-pseudotyped vector which carried human-bovine chimeric fibers, was able to bind to FX *in vitro*, but ineffective to promote liver transduction *in vivo* [39]. The fact that both HAdV5F35 and HAdV5F2/BAdV4 vectors carried the same hexon capsomere serotype and varied only in their fiber subtype incited us to explore the role of the fiber in the cell entry pathway and transduction mediated by HAdV5-based vectors in the presence of FX.

Our results confirmed that FX bound to HAdV5 hexon protein via its Gla-domain and enhanced the binding of serotype 5 hexon protein and HAdV5wt virus particles to surface-immobilized HS. We also found that FX promoted the interaction of HS with HAdV5F^TTAT^ and HAdV5Pb^EGD^ mutants *in vitro*, significantly enhanced the cell transduction by HAdV5wt, and was able to rescue the lower infectivity of HAdV5F^TTAT^ and HAdV5Pb^EGD^ mutants. Since FX contains a RGD tripeptide motif at position 227–229, it was conceivable that in the case of HAdV5Pb^EGD^ mutant, FX might compensate for the integrin binding defect of penton base EGD mutant. However, this hypothesis could be excluded for two reasons: (i) activated FX (FXa) which had lost the activation peptide (amino acid sequence 183–234) including the RGD motif, showed the same effect on HAdV5Pb^EGD^ infectivity as RGD-containing FX (data not shown); (ii) the activity of FX required the presence of HSPG at the cell surface, whereas RGD-dependent integrins seemed to be dispensable.

Intriguingly, however, we found that, although FX promoted the binding of fiber-pseudotyped vector HAdV5F35 to HS *in vitro* and to cellular HSPG *in vivo*, it failed to enhance, but instead decreased, the HAdV5F35-mediated transduction of CAR- and CD46-negative, but HSPG-positive CHO cells. It was recently hypothesized that the absolute levels of CAR, CD46, and HSPG on the surface of target cells would define the importance of FX in modulating cell binding and transduction mediated by HAdV5, HAdV35, and serotype 5/35 chimeric viruses: thus, in the presence of FX, the high affinity of serotype 35 fiber for CD46 would overcome the serotype 5 hexon∶FX interaction, resulting in reduced liver transduction by the chimeric vector HAdV5F35 *in vivo*, compared to HAdV5 [37]. However, this did not explain the limitation by FX of the transduction of CHO-CD46 cells *in vitro* by chimeric vectors carrying serotype 35 fibers, as previously reported [37], and observed in the present study (refer to Fig. 5), or the lower transduction of CD46 transgenic hepatocytes by a chimeric HAdV5/35 vector *in vivo* [38]. In the latter study, CD46-transgenic mice injected with a HAdV5/35-based chimeric vector carrying serotype 35 fiber knobs showed a two orders of magnitude lower liver transduction and 20-fold lower adenoviral genome content, compared to HAdV5-based vector [38].

Our analysis of the cell entry and trafficking pathway of HAdV5F35 vector in complex with FX provided some clues to reconcile these apparent contradictions, and to understand the possible mechanisms underlying the FX effect. We showed that FX augmented by 10-fold the attachment and cellular uptake of HAdV5F35, and confirmed that the negative effect of FX on HAdV5F35-mediated cell transduction did not result from a binding defect at the cell attachment step, but was due to intracellular mechanisms, as previously hypothesized [37]. According to our data, (i) the *primum movens* was the fiber 35-mediated addressing of HAdV5F35 particles (bound or unbound to FX) to the late endosomal/lysosomal compartment; (ii) FX-bound HAdV5F35 particles remained sequestered in this compartment, due to the higher stability of the FX-HAdV5F35 complexes in an acidic environment, which in turn (iii) delayed the trafficking of HAdV5F35 particles to the nucleus. (iv) An additional mechanism involved the exocytic pathway: following the cellular uptake of FX-HAdV5F35 complexes, significant amounts of exosome-associated HAdV5F35 were released in the extracellular milieu, a phenomenon which was not observed in the absence of FX. This was not observed with HAdV5wt either, with or without FX. Our results therefore addressed the puzzling question why, in contrast to HAdV5 vectors, fiber-chimeric vectors such as HAdV5F35 do not efficiently transduce liver cells after intravenous injection, although they contain the same HAdV5 hexons, which mediate binding to heparan sulfate on liver cells via blood FX. Our data suggested that the retention of HAdV5F35 particles in late endosomal compartments, triggered by HAdV35 fibers, was further enhanced by the hexon∶FX∶heparan sulfate interaction. This mechanism led to an abortion of HAdV5F35 transduction in the presence of FX, and most likely explained why HAdV5F35 vectors do not transduce liver cells after intravenous injection in mice.

The intracellular trafficking pathway of subspecies B HAdVs has been well studied [41], [58]. It has been shown that, despite high levels of binding to cells and similar internalization kinetics, fiber-pseudotyped or full serotype species B HAdVs remain in late endosomes or lysosomes for relatively long periods of time after infection, and take significantly longer than species C HAdV to reach the nucleus [41], [58]. This supposedly favored the recycling of the vectors to the cell surface and reduce their transduction efficiency, in contrast to HAdV5. Our experimental data on exosomal release of serotype 35 fiber-pseudotyped vector HAdV5F35 infection in the presence of FX supported the latter hypothesis. In conclusion, FX would be benefical to the infection of CAR-lacking cells by species C HAdVs, e.g. HAdV5, which transit via the early endosomal pathway, but detrimental to species B HAdVs, e.g. HAdV35, which follow the late endosomal pathway.

In the light of the acid stability of the FX-HAdV5F35 complex that we observed, it remained to explain the relative efficient transduction of CHO-CD46 cells by HAdV5F35 in the presence of FX (refer to Fig. 5). We assume that in the absence of CD46, such as in CHO-K1 cells, only the alternative receptors HSPG via the intermediate ligand FX would mediate the cell binding and entry of HAdV5F35, and all or nearly all vector particles would be complexed with FX, and retarded or trapped within the late vesicular compartment. In the presence of CD46 receptors however, e.g. in CHO-CD46 cells, FX became dispensable, and a significant number of free, FX-unbound HAdV5F35 particles would be endocytosed via the CD46 pathway. These free particles would normally escape the late endosomal vesicles and reach the nucleus, unlike FX-complexed HAdV5F35 particles.

Previous analyses of the intracellular fate of chimeric adenoviruses have shown that the fiber protein is the major determinant of the cell trafficking of incoming virions [57], [58], [81]. Thus, the targeting of HAdV5 pseudotyped with fibers of serotype 7 (a serotype belonging to species B HAdV) to the lysosomal pathway is under the control of the fiber serotype [57]. The present study provided another example of dominant effect of species B fiber, serotype 35 fiber, which occurred in the absence of CD46 and any other cognate fiber receptor at the plasma membrane. The endocytosis and retention of FX-HAdV5F35 complex in the late endosomal compartment, characteristic of species B HAdVs [41], [58], implied that the nature of the adenoviral fiber was dominant over the high affinity serotype 5 hexon∶FX interaction as a determinant of the choice of the endocytic compartment and intracellular trafficking pathway of HAdV particles. This confirmed previous reports on the intracellular functions associated with the fibers carried by incoming adenovirions [57], [58], [70], [81], [82]. The molecular mechanism and factors involved in the cellular traffic determinism of adenoviral fiber remains an open question, but should be taken into consideration in the future design of target tissue-redirected adenoviral vectors.

## Materials and Methods

### Cell lines

E1A-E1B-trans-complementing HEK-293 cell line (abbreviated 293; CRL 1573) was obtained from the American Type Culture Collection (Manassas, Va). HEK-293 cells were cultured as monolayers in DMEM supplemented with 10% fetal calf serum (FCS, Sigma), penicillin (200 U/ml), and streptomycin (200 µg/ml; Gibco-Invitrogen) at 37°C and 5% CO_2_. The 293-derived, fiber-trans-complementing cell line, abbreviated 293-Fiber, was obtained from Transgene SA (Strasbourg, France). 293-Fiber cells were grown in the same medium as 293 cells, supplemented with hygromycin at 350 µg/ml [70], [83]. Chinese hamster ovary cells CHO-K1, and proteoglycan-deficient CHO-2241 cells (pgsB-618, ATCC code CRL-2241) were obtained from the European Collection of Cultured Cells via the Institut de Biologie Structurale, Grenoble, France [79]. CAR-expressing CHO cells (CHO-CAR) were obtained from Dr. J. Bergelson [4], and CD46-expressing CHO cells (CHO-CD46) from Dr. D. Gerlier [84]. CHO-K1, CHO-2241, CHO-CAR and CHO-CD46 cells were cultured as monolayers in Alpha-MEM supplemented with 10% FCS, penicillin and streptomycin as above (Gibco-Invitrogen). All cells were incubated at 37°C under 5% CO_2_ .

### HAdV5-based vectors and nomenclature

Replication-deficient HAdV5 vectors (E1A–E1B, and E3 deleted) expressing GFP were propagated in *trans*-complementing cell line 293. Their genetic construction and phenotypes have been described in detail in previous studies [85]–[93]. Since all the vectors used in the present study contained the same reporter gene coding for the green fluorescent protein (EGFP) cloned downstream to the hCMV promoter, their acronyms did not specify this reporter gene, in contrast to our previous studies. For reason of simplification, their acronyms only referred to the capsid component serotype and to the protein mutated, penton base (Pb) or fiber (F).

#### (i) HAdV5 vector with wild-type (wt) capsid

Control HAdV5 vector with nonmodified capsid was abbreviated HAdV5wt.

#### (ii) Penton base mutant

Mutant HAdV5Pb^EGD^ carried a RGD-to-EGD substitution at position 340 in the penton base coding sequence.

#### (iii) Fiber shaft substitution mutant

In the HAdV5F^TTAT^ vector, the KKTK motif at position 91–94 in the fiber shaft domain was modified into 91-TATT-94 using conventional PCR-based mutagenesis [88], [91].

#### (iv) Chimeric fiber vector HAdV5F35

HAdV5F35 carried the HAdV serotype 35 fiber knob and shaft domains fused to the HAdV5 fiber tail [68], [85].

### Adenovirus purification and titration

HAdV5-based vectors were purified by CsCl gradient ultracentrifugation using conventional methods [85], [94]. The infectious titer of the HAdV5 vector stocks was determined by the plaque assay method in HEK-293 or in double *trans*-complementing cell line 293-Fiber [70] for HAdV5 fiber mutants, and expressed as PFU per ml [91], [92]. The titer in physical particles (viral particles; vp) was determined by absorbance measurement at 260 nm (*A*_260_) of 1-ml samples of SDS-denatured virions (0.1% SDS for 1 min at 56°C) in a 1-cm-path-length cuvette, using the respective formula: *A*_260_ of 1.0 = 1.1×10^12^ vp/ml for HAdV5 (genomic DNA = 36 kbp). HAdV5 particle titers ranged from 5×10^11^ to 1×10^12^ vp/ml, with infectious titers between 2×10^10^ and 5×10^10^ PFU/ml [95].

### Fluorescent labeling of adenovirus particles and FX protein

Alexa Fluor® 488 dye (tetrafluorophenyl ester; Molecular Probes; Invitrogen), and Alexa Fluor® 555 dye (succinimidyl ester; Molecular Probes; Invitrogen), were abbreviated Alexa-488 and Alexa-555, respectively. Random labeling of HAdV5wt or HAdV5F35 vector particles with Alexa-488 was carried out as follows. Aliquots of 1×10^12^ vector particles in suspension in 0.05 M HEPES buffer pH 7.2 (900 µl, final volume) were incubated with chemically reactive Alexa-488 used at a 20-fold excess over the 18,000 amino groups present at the surface of the adenoviral capsid, for 2 h at room temperature (RT) and in argon atmosphere. The reaction was stopped by adding 100 µl 1 M lysine solution in 0.05 M HEPES buffer pH 7.2, corresponding to a 5-fold excess of lysine over the Alexa-488 reagent. After 1 h incubation at RT, the labeled-vector particles were separated from unreacted dye by gel filtration on a PD10 column. For labeling of FX protein with Alexa-555, FX protein (5 mg/ml) was reacted with 1 mM Alexa Fluor® 555 dye in 0.05 M HEPES buffer pH 7.2, for 1 h at RT, and excess of unreacted dye eliminated by dialysis against PBS.

### Live cell imaging and time-lapse microscopy

Samples of CHO-K1 or CHO 2241 cell monolayers (5×10^5^ cells/well) were seeded directly onto poly L-lysine-coated glass dishes (Mattek Corp., USA), and maintained in the appropriate medium at 37°C for 24 h. Cells were infected with 35 µl of recombinant baculovirus suspension expressing fluorescent markers of cellular organelles or compartments (CellLight™ Lysosomes-RFP and CellLight™ Endosomes-RFP *BacMam 2.0*) per cm^2^ of monolayer in serum-free medium for 4 h at RT, according to the supplier instructions (Invitrogen). Suspension was then withdrawn and replaced by culture medium supplemented with 10% FCS. Cell samples were further incubated overnight at 37°C under 5% CO_2_ atmosphere, then processed for infection with fluorescent-labeled vector particles, as follows. The following day, the cells were washed with cold PBS (4°C), and overlaid with 1 ml fresh and cold (4°C) medium. Alexa-488-labeled vector particles (10,000 vp/cell, in a volume of 100 µl) were added to the medium, and incubated on ice for 1 h. The cells were then washed 3 times with PBS, followed by fresh and cold (4°C) medium. Cell samples were kept on ice until they were transferred to 37°C in the incubation chamber of the confocal microscope. Observations were performed from 10 min to 3 h pi, using an inverted confocal laser scanning microscope (LSM 510 Meta; Carl Zeiss, IAB), equipped with a 63× oil immersion objective (Plan-Apochromat 63×/1.4) and a humidified CO2- and temperature-controlled incubation chamber. The Alexa-488 dye was excited by the Argon laser and the emission was collected with a BP 500–550 nm filter. Orange channel used 543 nm HeNe laser excitation and LP560 emission filter. The images were mainly collected in the focal plane crossing the cell nucleus with a resolution of 512×512 pixels (Zoom 1) and 4× line average. The collection time was 3 s per channel. The pinhole of the fluorescence channels was set to one Airy unit corresponding to the optical section of less than 1 µm and the signal intensity was adjusted with a PMT gain to completely fill the detector dynamic range. The two channels were acquired in a sequential mode to avoid spectral cross-talk, and the acquisition of transmitted light image was concomitant with the green channel.

### Flow cytometry

Aliquots of cells (2×10^5^ per well) were seeded in fully supplemented appropriate medium into 24-well plates. 24 h later, the cells were washed three times with PBS, and 500 µl of appropriate medium containing 1% penicillin/streptomycin and 10% glutamine but no FCS was added. For transduction assays, different vector preparations were added to the medium at various MOI, with or without incubation with FX at different concentrations. After 2 h, the cells were extensively washed with PBS prewarmed at 37°C, then 1 ml of fresh fully supplemented medium was added and cells further incubated at 37°C for an additional 22 h. Cells were resuspended in PBS and analyzed by flow cytometry, using a FACSCanto™ II cytometer (Becton Dickinson Biosciences). 20,000 events were acquired for each sample and the results were analyzed using the DIVA 6 software (Becton Dickinson).

### Surface plasmon resonance (SPR) assays

The source of naturally hypersulfated heparin (HS), sodium salt, was the porcine intestinal mucosa (Sigma). HS calibrated to 9 kDa was biotinylated and immobilized on a CM4 BIAcore sensorchip (GE Healthcare, Saclay, France), using a BIAcore3000 (GE Healthcare), as previously described [48], [49]. Two flowcells were prepared by sequential injections of EDC/NHS, streptavidin, and ethanolamine. One of these flowcells served as negative control, while biotinylated heparin was injected on the other one, to get an immobilization level of 80–90 response units (RU). All SPR experiments were performed using HBS buffer (10 mM HEPES, 150 mM NaCl, pH 7.4) supplemented by 3 mM CaCl_2_, at a flow rate of 5 µl/min. Interaction assays involved injections of different amounts of protein or virus over the heparin-coated and negative control surfaces, followed by a 3-min dissociation time with buffer. At the end of each cycle, the heparin surface was regenerated by injections of 2 M NaCl (2 min). Sensorgrams shown corresponded to on-line subtraction of the negative control to the heparin surface signal.

### Preparation of plasma membrane-shedded microvesicles (MVs) and MVB-released exosomes (EXOs)

#### (i) Source of MVs and EXOs

Confluent CHO-K1 cells were transduced with HAdV5wt or HAdV5F35 at MOI 5,000, with or without FX, and harvested at 2 h and 24 h pi. The cell culture medium was harvested and floating cells were pelleted by centrifugation at 2,000× *g* for 10 min. The supernatant was further clarified from cell debris by centrifugation for 2 min at 13,000× *g* and 4°C. This final supernatant (S0) was the source of plasma membrane-derived MVs and EXOs, prepared as described in [96].

#### (ii) Isolation of MV

Fraction S0 was centrifuged for 2 h at 30,000× *g* and 4°C, and pellet P1 was saved: it mainly contained MVs.

#### (iii) Isolation of EXOs

Supernatant S1 was further centrifuged at 100,000× *g* and 4°C for 2 h, giving supernatant S2, which was discarded, and pellet P2, which contained EXOs. Pellets were resupended in PBS and subjected to DNA extraction, using QIAamp DNA Blood Mini kit (Quiagen).

### Quantification of AdV vector genomes by real-time PCR and real-time RT-PCR

HAdV5 vectors which were possibly carried over with MVs were detected and quantitated by real-time PCR, using HAdV5 or HAdV35 specific primers and probe selected from the fiber gene. Extraction of DNA was carried out using the QIAamp DNA Blood Mini Kit (Quiagen) and real time PCR reactions were carried out using the LightCycler DNA Master SYBR Green I kit (Roche) and the LightCycler instrument (Roche). For the serotype 5 fiber gene, the primers used were: sense 5′-GCTACAGTTTCAGTTTTGGCTG-3′ and antisense 5′-GTTGTGGCCAGACCAGTCCC-3′ ; for the serotype 35 fiber gene, the primers used were : sense 5′- TGGCTTCACACAAAGCCCAGACG-3′ and antisense 5′- ACACGTAGCCATTAACAAGCCCTCC-3′. As internal control, ß-actin gene was amplified using the following primers, sense 5′-GCTGTGTTCTTGCACTCCTTG-3′ and antisense 5′- CGCACGATTTCCCTCTCAGC-3′.

### Proteins

FX, activated FX (FXa) which lacked the RGD motif contained in the activation peptide [97], and the truncated form FXGL (Gla domainless) were all purchased from CRYOPREP (Montpellier, France). Proteolytically inactive human FX, blocked with Dansyl-EGR (FX-DEGR), was also purchased from CRYOPREP. HAdV5wt hexon, penton (penton base + fiber) and fiber proteins were isolated from lysates of HAdV5wt-infected 293 cells [85]. HAdV5wt penton base and penton base RGD-340-EGD mutant, were recombinant proteins isolated from recombinant baculovirus-infected cells [98], [99]. Viral proteins were purified according to a conventional protocol adapted to fast protein liquid chromatography [100]–[103]. Protein samples were analyzed by sodium dodecyl sulfate polyacrylamide gel electrophoresis (SDS-PAGE) and western blotting, as previously described [85].

### Adenovirus type 3 penton dodecahedron (Pt-Dd) purification, labeling and intracellular trafficking

HAdV3 Pt-Dd was produced in recombinant baculovirus-infected insect cells coexpressing HAdV3 penton base and fibre proteins, as previoulsy described [40], [77]–[79]. HAdV3 Pt-Dd was purified by sucrose gradient density, and dialysed against HEPES-NaCl buffer (20 mM HEPES, pH 7.4, 150 mM NaCl). Fluorescent-labeled Pt-Dd was obtained by incubation of a Pt-Dd protein solution at 1 mg/ml with Cy5-monoNHS-Ester at 1 mM (GE Healthcare, PA15101) for 2 h at room temperature, followed by extensive dialysis against PBS to remove unbound dye. Cy5-labeled Pt-Dd (10 µg/ml) was incubated at 37°C for 10 min to 2 h with CHO-CD46 cells transduced one day before with recombinant baculovirus suspension expressing the Lamp1 fluorescent marker of the late endosome/lysosome compartment (CellLight™ Lysosomes-RFP *BacMam 2.0*; Invitrogen), as described above. After removal of Cy5-labeled Pt-Dd and rinsing the cell monolayer with prewarmed medium, live imaging was performed using the LSM 510 Meta inverted confocal laser scanning microscope (Carl Zeiss, IAB) as above, with the RFP and Cy5 filters.

### Electron microscopy (EM)

Specimens were processed for EM and observed as previously described [104], [105]. In brief, cells were harvested at 2 h pi, pelleted, fixed with 2.5% glutaraldehyde in 0.1 M phosphate buffer, pH 7.5, post-fixed with osmium tetroxide (2% in H20) and treated with 0.5% tannic acid solution in H20. The specimens were dehydrated and embedded in Epon (Epon-812; Fulham, Latham, NY). Ultrathin sections were stained with 2.6% alkaline lead citrate and 0.5% uranyl acetate in 50% ethanol, and post-stained with 0.5% uranyl acetate solution in H_2_O. Grids were examined under a Jeol JEM-1400 electron microscope, equipped with an ORIUS™ digitalized camera (Gatan France, 78113-Grandchamp). For statistical EM analyses, a minimum of 50 grid squares containing 5 to 10 cell sections each were examined for counting virions in different cell compartments.

### Statistics

Results were expressed as mean ± SEM. of n observations. Sets of data were compared with an analysis of variance (ANOVA) or a Student's *t* test. Differences were considered statistically significant when *P*<0.05. Symbols used in figures were (*) for *P*<0.05, (**) for *P*<0.01, (***) for *P*<0.001, and ns for no significant difference, respectively. All statistical tests were performed using GraphPad Prism version 4.0 for Windows (Graphpad Software).
